# Intracluster correlation coefficients for neonatal trials: an analysis of national population level clinical data

**DOI:** 10.1186/s13063-026-09893-z

**Published:** 2026-07-23

**Authors:** S. Jawad, A. T. Prevost, C. Gale

**Affiliations:** 1 Neonatal Medicine, School of Public Health, Faculty of Medicine, Imperial College London, Chelsea and Westminster Hospital Campus, 369 Fulham Road, London, SW10 9NH UK; 2 Moorfields Eye Hospital NHS Foundation Trust, 162 City Road, London, EC1V 2PD UK; 3 Cicely Saunders Institute of Palliative Care, Policy and Rehabilitation, King’s College London, Bessemer Road, London, SE5 9PJ UK; 4 Centre for Paediatrics and Child Health, Imperial College, London, UK

**Keywords:** Intracluster correlation coefficient, Neonatal trial design, Design effect, Cluster, Randomised controlled trial, Preterm, Neonatal

## Abstract

**Background:**

Clustering of outcomes occurs naturally in neonatal data due to multiple births clustered by mother and infants clustered within the neonatal unit administering their care. Estimating these cluster effects is important for neonatal study design and impacts upon sample sizes for neonatal trials.

**Methods:**

We analysed retrospective data from all neonatal admissions in England and Wales between January 2016 and January 2020 held in the National Neonatal Research Database. Intracluster correlation coefficients (ICCs) and their 95% confidence intervals were calculated for core neonatal outcomes and those commonly used in trials, at the level of both the neonatal unit and mother. Results were stratified by gestational age and neonatal unit level of birth. To illustrate the impact of clustering, the design effect was estimated for a theoretical cluster trial.

**Results:**

Intracluster correlation coefficients varied between outcomes and gestational age groups. Neonatal unit level intracluster correlation coefficients were low for mortality (0.0054, 95% CI 0.0039, 0.0068) and other core outcomes (severe necrotising enterocolitis 0.0042, 95% CI 0.0020, 0.0063) and were higher for outcomes related to care delivery (duration of intensive care 0.0237, 95% CI 0.0177, 0.0298; duration receiving parenteral nutrition 0.0265, 95% CI 0.0197, 0.0332). Gestation at birth was inversely correlated with neonatal unit ICC estimates. At the level of the mother, ICC estimates were generally larger, especially for preterm infants.

**Conclusions:**

We have estimated ICCs for key neonatal outcomes at the level of both neonatal unit and mother with high precision using national, population-level routinely recorded data. These ICC estimates can be used to inform future neonatal studies and sample size calculations for neonatal trials.

**Table:** 

Step-by-step guide on how to use these ICCs
1) Identify the gestational age stratum which is closest to the planned trial population
2) Specify whether neonatal unit or mother clustering is of interest
3) Identify and match the outcome for which the ICC is needed (usually the primary outcome)
4) Choose the corresponding ICC estimate and consider using the upper confidence interval estimate for a more conservative approach
5) Inflate the sample size using the ICC and if unequal sample sizes are expected

## Background

Cluster trials are the optimal design to address certain clinical research questions, and in some situations, may be the only feasible approach—for example where an intervention can only be administered at the level of the neonatal unit [1], or for individual level interventions when contamination between trial participants risks adherence to the randomised treatment: for example a novel mobile phone app allocated to parents as an intervention but which may also be used by parents allocated to the standard care arm.

Cluster effect estimates such as intracluster correlation coefficients (ICC) are critical to accurately calculate sample size and to aid in choosing an appropriate analysis but are lacking in neonatal care [2]. Where they have been reported, cluster effect estimates are commonly obtained from previous trials or observational studies; however, this approach has limitations: the population, outcomes and treatment arms may not match that of the proposed trial and sample sizes are commonly low, leading to inaccuracies and imprecision [3]. This can lead to mis-specified cluster effects, inaccurate sample size estimates, loss of statistical power in the subsequent trial, and ultimately to research waste [4]. 

All neonatal trials (both cluster and individually randomised) are further complicated by the presence of clustering at multiple additional levels. Neonatal units may be nested within regional neonatal networks or federated health systems, while infants belonging to multiple births are clustered at the level of the mother. Multiple births comprise a considerable proportion (up to 30%) of participants in preterm trials [5], and unblinded individually randomised neonatal trials commonly randomise infants from multiple births together recognising the preference of parents and patients for this approach [6]. Consequently, many individually randomised neonatal trials require cluster estimates at the level of mothers to compute accurate sample sizes and plan appropriate analyses.

The aim of this study is to calculate cluster estimates with high precision at the level of both the neonatal unit and the mother using routinely collected, population-level, observational data from the UK. This population-level analysis has been conducted in other areas such as primary care [7] but not, to the best of our knowledge, in neonatal care.

## Methods

We used the National Neonatal Research Database (NNRD) as the sole source of data. The NNRD [8] holds data from infants admitted to all National Health Service (NHS) neonatal units in England, Scotland and Wales (approximately 90,000 infants annually). The included population comprised babies admitted for neonatal care at a contributing neonatal unit without restriction by gestational age at birth. Data are extracted from neonatal electronic health records completed by health professionals during routine clinical care. A defined dataset, the Neonatal Dataset [9] of approximately 450 data items, is extracted quarterly, cleaned, and duplicates and queries about discrepant and implausible data are fed back to health professionals and rectified. Completeness and accuracy of NNRD data have been validated [8] in comparison to independent trial case report forms. Deidentified data used in this study were taken from all neonatal admissions, including any transfers to other neonatal units, between January 2016 and January 2020 inclusive. The neonatal data analysis unit (NDAU) holds UK Research Ethics Committee approval (21/LO/0024) for the NNRD.

Neonatal units in the UK are organised in regional networks of non-tertiary and tertiary neonatal units. The intention is for infants requiring high level care to receive it in a tertiary neonatal unit, with step-down transfer to a non-tertiary neonatal unit within the same network when appropriate. Neonatal units in English hospitals are classified as special care baby units (SCBU), local neonatal units (LNU) and tertiary level neonatal intensive care units (NICU) [10]. These levels and designations are analogous with but differ slightly from those of the American Academy of Paediatrics [11]. Neonatal intensive care units provide ongoing intensive care for babies of all gestations and may offer surgical and other specialist care such as cardiac surgery; local neonatal units are generally expected to be able to care for uncomplicated, singleton infants from 27 weeks’ gestation onwards and twins or higher order multiples from 28 weeks’ gestation onwards. Special care baby units are expected to provide care for babies from 32 weeks’ gestation and provided birthweight is above 1000 g but are not expected to provide intensive care apart from initial stabilisation for transfer.

We calculated cluster effect estimates at two levels:

1. At the level of the neonatal unit: clusters were defined as the first provider of neonatal care.
2. At the level of the mother for multiple births: clusters were identified as infants with the same mother identifier, birth year and gestational age.

We calculated cluster effect estimates for outcomes identified as critically important by the Core Outcomes in Neonatology (COIN) [12] project where NNRD data were available, and for additional outcomes commonly reported in neonatal clinical trials [13]. The following outcomes were included:

1. Survival, defined as alive at final neonatal unit discharge.
2. Necrotising enterocolitis (NEC) Bell’s Stage II or greater, defined using the National Neonatal Audit Program (NNAP) definition for infants born less than 32^+0^ gestation weeks^+days^ and who survived to at least 48 h after birth. NEC was defined as a diagnosis at surgery, post-mortem or on clinical or radiographic signs [14].
3. Severe necrotising enterocolitis (NEC), defined as a diagnosis of NEC and surgery for NEC or death caused by NEC [15].
4. Brain injuries were defined separately for term and preterm infants. Infants were defined as having a brain injury if they were diagnosed with intracranial haemorrhage, perinatal stroke, hypoxic ischaemic encephalopathy, central nervous system infection, kernicterus (bilirubin encephalopathy) or a recorded seizure. In addition, preterm infants (< 37 gestational weeks at birth) also had brain injury if they had any evidence of preterm white matter disease (periventricular leukomalacia) [16].
5. Death or bronchopulmonary dysplasia (BPD) defined according to the NNAP definition [14] for infants born less than 32^+0^ gestation weeks^+days^.
6. Number of days in intensive care, determined using the BAPM 2011 levels of care [17].
7. Number of days receiving invasive ventilation defined as the number of days where a baby received conventional ventilation, high-frequency oscillation ventilation or nitric oxide, delivered via endotracheal tube or tracheostomy.
8. Number of days receiving parenteral nutrition.
9. Number of days with central line in situ, defined as the number of days with any type of central vascular line in situ.
10. Number of days on the neonatal unit, defined as the number of days between admission and first discharge to a non-neonatal care setting among survivors only.

Intracluster correlation coefficients and asymptotic 95% confidence intervals were estimated from unadjusted one-way analysis of variance models using STATA’s (Stata Corp) “loneway” command for the neonatal unit clusters and multiple births clusters separately. Negative lower bound confidence intervals and negative ICC estimates were truncated to zero as the ICC is a nonnegative value. The ICC estimates for binary outcomes are specifically observed-scale estimates as opposed to latent-scale logistic ICC estimates. The ICCs for multiple births were estimated using the entire dataset including both singletons and multiples. A combination of gestational age and levels of care were used as stratification factors. These were chosen based on typical trial populations so that the cluster estimates reflect these populations; stratification groups are not mutually exclusive. ICCs for binary and continuous outcomes were calculated in the same way but are presented separately for the reader’s benefit. Descriptive statistics such as means, medians, 25th and 75th centiles were presented for all outcomes [18]. The ICC analyses were conducted using cases with non-missing outcome data. The NEC diagnoses were grouped into three categories: (1) confirmed NEC, (2) no NEC (survival without NEC or no NEC but died prior to discharge home), (3) missing NEC data (died prior to discharge home but NEC diagnosis data missing or NEC diagnosis data missing). Bronchopulmonary dysplasia (BPD) missing values were defined as indeterminable BPD. Receipt of respiratory support at time of discharge was used to determine BPD for infants discharged before 36 weeks postmenstrual age.

A trial scenario was created to illustrate the design effect of different ICCs for individual and cluster randomised trials. Design effects (also known as variance inflation factors) are defined as Deff=1+(n-1)ρ, where n is the mean cluster size and ρ the ICC [19]. The design effect and the sample size are multiplied in order to inflate the sample size to be appropriately powered for a cluster design. The design effect was firstly calculated at the level of the mother, assuming a study with both singletons and multiples would have an approximate average cluster size of 1.1 infants. This average cluster size was calculated from the data. A second design effect was estimated for the neonatal unit level. Here, the average cluster size was assumed to be 40 trial participants per neonatal unit, estimated from the typical number of neonatal admissions in the UK. A final design effect for neonatal unit cluster randomised trials was produced as a product of the mother and neonatal unit level design effects [20]. The stated design effect is a simple formula for parallel cluster and equal cluster sizes and requires further modification when used with alternative cluster designs such as stepped-wedge, cluster-crossover and repeated-period designs.

## Results

Routinely recorded data from babies born between January 2016 and January 2020 from 204 neonatal units (some neonatal units were restructured over the study period) and 417,675 babies were included in this analysis.

Cluster estimates at the level of the neonatal unit for both continuous and binary outcomes are reported in Table 1 for dichotomous outcomes and Table 2 for continuous outcomes. Cluster estimates vary between outcomes: dichotomous outcomes corresponding to the neonatal core outcome set have lower ICC values (such as survival for babies of all gestations 0.0054 95% CI (0.0039, 0.0068)) (Table 1) than continuous outcomes describing delivery or care (such as days on neonatal unit (0.0450 95% CI (0.0338, 0.0562)) (Table 2). The ICC varied within each outcome between the gestational age and level of care groups. The ICC for term infants tended to be smaller than the ICC for more preterm infants. The ICC for the combined outcome of BPD or death had the reverse relationship: ICC increased with gestational age (Table 1). Cluster estimates calculated at the level of the mother are reported in Supplemental Tables 1 and 2; these were higher than neonatal unit level ICC.

**Table 1:** Cluster estimates at level of the neonatal units for binary outcomes, data from National Neonatal Research Database 2016-2020

	Missing outcome data n (%)^a^	*N*	Mean cluster size	Outcome prevalence *n* (%)	Within cluster SD	ICC	ICC 95% confidence interval
Survival (*N*=417,675) ^b^							
All infants	0 (0)	417,675	2,047.43	413,405 (99.0)	0.10	0.0054	(0.0039, 0.0068)
Gestational age and level of care groups							
<28 weeks, initial care in NICU	0 (0)	6,752	143.66	5,394 (79.9)	0.40	0.0189	(0.0081, 0.0297)
<32 weeks, initial care in NICU or LNU	0 (0)	27,331	224.02	25,070 (91.7)	0.27	0.0126	(0.0080, 0.0173)
32–36 weeks, initial care in NICU, LNU or SCBU	0 (0)	119,105	583.85	118,421 (99.4)	0.08	0.0019	(0.0011, 0.0026)
≥37 weeks, initial care in NICU, LNU or SCBU	0 (0)	265,014	1,299.08	264,131 (99.7)	0.06	0.0026	(0.0018, 0.0035)
Necrotising enterocolitis (*N*=32,528) ^b^							
Gestational age and levels of care groups							
<28 weeks, initial care in NICU	1,250 (19.50)	5,160	109.79	609 (11.80)	0.32	0.0116	(0.0030, 0.0202)
28–31 weeks, initial care in NICU or LNU	3,801 (20.71)	14,551	119.27	433 (2.98)	0.17	0.0060	(0.0023, 0.0097)
<32 weeks, initial care in NICU or LNU	5,347 (20.07)	21,298	174.57	1,205 (5.66)	0.23	0.0079	(0.0043, 0.0115)
Severe necrotising enterocolitis (*N*=33,370) ^b^							
Gestational age and levels of care groups							
<28 weeks, initial care in NICU	-	6,752	143.66	585 (8.66)	0.28	0.0062	(0.0007, 0.0116)
28–31 weeks, initial care in NICU or LNU	-	18,539	151.96	237 (1.28)	0.11	0.0008	(0.0000, 0.0027)
<32 weeks, initial care in NICU or LNU	-	27,331	224.02	978 (3.58)	0.19	0.0041	(0.0018, 0.0063)
Brain injury (*N*=416,977) ^b^							
Gestational age and levels of care groups							
<28 weeks, initial care in NICU	-	6,751	143.64	1,337 (19.80)	0.40	0.0119	(0.0040, 0.0197)
<32 weeks, initial care in NICU or LNU	-	27,328	224.00	2,807 (10.27)	0.30	0.0077	(0.0044, 0.0109)
32–36 weeks, initial care in NICU, LNU or SCBU	-	119,013	583.40	1,303 (1.09)	0.10	0.0016	(0.0009, 0.0023)
≥37 weeks, initial care in NICU, LNU or SCBU	-	264,595	1,297.03	5,323 (2.01)	0.14	0.0078	(0.0055, 0.0102)
Death or bronchopulmonary dysplasia (*N*=31,805) ^b^							
Gestational age and levels of care groups							
<28 weeks, initial care in NICU	36 (0.58)	6,218	132.30	4,737 (76.18)	0.42	0.0108	(0.0032, 0.0184)
28–31 weeks, initial care in NICU or LNU	98 (0.55)	17,767	145.63	3,503 (19.72)	0.39	0.0195	(0.0126, 0.0264)
<32 weeks, initial care in NICU or LNU	148 (0.57)	25,900	212.30	9,534 (36.81)	0.47	0.0440	(0.0306, 0.0574)

N.B. Gestational age and care level strata are not mutually exclusive groups

*NICU* neonatal intensive care unit, *LNU* local neonatal unit, *SCBU* special care baby unit

^a^ Fields indicated by “- “refer to data collected via a checkbox indicating presence of condition. Therefore, it is not possible to distinguish between missing data and presence of no condition

^b^ Analysis includes 47 NICU units, 75 LNU units and 37 SCBU units, where n is the number of infants with non-missing outcome data and neonatal unit data

**Table 2:** Cluster estimates at level of the neonatal unit for continuous outcomes, data from National Neonatal Research Database 2016-2020

	Missing outcome data N (%)^1^	*N*	Summary statistics			Within cluster SD	ICC	ICC 95% confidence interval
			Mean cluster size	Min, Max	Median (25^th^, 75^th^ centile)			
Days receiving intensive care (*n*=417,096) ^2^								
All infants	-	417,096	2,044.59	[0, 321]	0 (0, 0)	6.77	0.0237	(0.0177, 0.0298)
Gestational age and levels of care groups								
<28 weeks, initial care in NICU	-	6,751	143.64	[0, 277]	19 (11, 36)	23.58	0.0353	(0.0178, 0.0529)
<32 weeks, initial care in NICU or LNU	-	27,328	224.00	[0, 321]	8 (3, 16)	17.75	0.0377	(0.0261, 0.0494)
32–36 weeks, initial care in NICU, LNU or SCBU	-	119,013	583.40	[0, 254]	0 (0, 0)	4.08	0.0120	(0.0090, 0.0150)
≥37 weeks, initial care in NICU, LNU or SCBU	-	264,592	1,297.02	[0, 180]	0 (0, 0)	2.42	0.0177	(0.0126, 0.0228)
Days receiving invasive ventilation (*n*=417,096) ^2^								
All infants	-	417,096	2,044.59	[0,297]	0 (0, 0)	4.90	0.0156	(0.0115, 0.0196)
Gestational age and levels of care groups								
<28 weeks, initial care in NICU	-	6,751	142.44	[0, 273]	10 (3, 26)	20.19	0.0400	(0.0206, 0.0595)
<32 weeks, initial care in NICU or LNU	-	27,328	227.61	[0, 297]	1 (0, 6)	14.21	0.0352	(0.0242, 0.0462)
32–36 weeks, initial care in NICU, LNU or SCBU	-	119,013	646.81	[0, 237]	0 (0, 0)	2.62	0.0065	(0.0048, 0.0083)
≥37 weeks, initial care in NICU, LNU or SCBU	-	264,592	1,438.00	[0, 175]	0 (0, 0)	1.84	0.0095	(0.0067, 0.0123)
Days receiving any parenteral nutrition (*n*=417,096) ^2^								
All Infants	-	417,096	2,044.59	[0, 283]	0 (0, 0)	6.16	0.0265	(0.0197, 0.0332)
Gestational age and levels of care groups								
<28 weeks, initial care in NICU	-	6,751	142.44	[0, 204]	15 (9, 25)	20.67	0.0397	(0.0204, 0.0590)
<32 weeks, initial care in NICU or LNU	-	27,328	227.61	[0, 283]	9 (5, 15)	15.59	0.0411	(0.0285, 0.0536)
32–36 weeks, initial care in NICU, LNU or SCBU	-	119,013	646.81	[0, 250]	0 (0, 0)	4.36	0.0295	(0.0227, 0.0364)
≥37 weeks, initial care in NICU, LNU or SCBU	-	264,592	1,438.00	[0, 183]	0 (0, 0)	1.91	0.0202	(0.0144, 0.0259)
Days with central line in-situ (*n*=417,096) ^2^								
All infants	-	417,096	2,044.59	[0,314]	0 (0, 0)	6.59	0.0264	(0.0196, 0.0331)
Gestational age and levels of care groups								
<28 weeks, initial care in NICU	-	6,751	142.44	[0, 211]	15 (10, 26)	21.62	0.0448	(0.0235, 0.0661)
<32 weeks, initial care in NICU or LNU	-	27,328	227.61	[0, 314]	9 (4, 15)	16.50	0.0450	(0.0313, 0.0586)
32–36 weeks, initial care in NICU, LNU or SCBU	-	119,013	646.81	[0, 250]	0 (0, 0)	4.84	0.0204	(0.0155, 0.0253)
≥37 weeks, initial care in NICU, LNU or SCBU	-	264,592	1,438.00	[0, 191]	0 (0, 0)	2.40	0.0193	(0.0137, 0.0248)
Days on neonatal unit (*n*=413,404) ^2^								
All infants	2,180 (0.53%)	411,224	2,015.80	[0, 857]	3 (2, 9)	18.53	0.0450	(0.0338, 0.0562)
Gestational age and levels of care groups								
<28 weeks, initial care in NICU	299 (4.85%)	5,149	109.55	[0, 369]	95 (76, 116)	34.39	0.0357	(0.0171, 0.0542)
<32 weeks, initial care in NICU or LNU	794 (10.72%)	24,411	200.09	[0, 370]	52 (37, 78)	33.09	0.0673	(0.0480, 0.0867)
32–36 weeks, initial care in NICU, LNU or SCBU	706 (2.77%)	117,715	577.03	[0, 325]	9 (3, 16)	11.28	0.0365	(0.0282, 0.0449)
≥37 weeks, initial care in NICU, LNU or SCBU	652 (0.25%)	263,478	1,291.56	[0, 857]	2 (1, 5)	6.17	0.0341	(0.0245, 0.0437)

*NICU* neonatal intensive care unit, *LNU* local neonatal unit, *SCBU *special care baby unit

^1^Fields indicated by “- “refer to data collected via a checkbox indicating presence of condition. Therefore, it is not possible to distinguish between missing data and presence of no condition

²Analyses include 47 NICUs, 75 LNUs and 37 SCBUs, where n is the number of infants with non-missing outcome data and neonatal unit data

N.B. Gestational age and care level strata are not mutually exclusive groups

To illustrate the impact of different cluster estimates on a theoretical trial, design effects at mother and neonatal unit level are presented in Table 3. The design effect indicates the multiplier to be applied to the sample size calculation to account for clustering. As expected, the design effects differ in magnitude by outcome and by gestational age and care level. Mother-level design effects, in these examples, were all between 1.00 and 1.08 using an average cluster size of 1.1. Neonatal unit-level design effects were larger, ranging from 1.1 to 3.6 for a theoretical cluster trial with an average cluster size of 40. Design effects accounting for clustering at both levels again varied depending on the population of interest and ranged between 1.1 and 3.9 (Table 3).

**Table 3:** Design effects for each cluster level and for the combined clustering effect using a theoretical cluster size of 40 infants per neonatal unit and 1.1 infants per mother; design effect is defined using the average cluster size and ICC and is multiplied by the sample size in order to calculate an adequately powered sample size for a cluster randomised trial

	Mother level			Neonatal unit level			
	ICC	Mean cluster size	Design effect	ICC	Mean cluster size	Design effect	Design effect for randomising neonatal units with the presence of multiple births
Days on neonatal unit							
All infants	0.5714	1.1	1.057	0.0450	40	2.755	2.912
< 28 weeks, initial care in NICU	0.6178	1.1	1.062	0.0357	40	2.392	2.540
< 32 weeks, initial care in NICU or LNU	0.7762	1.1	1.078	0.0673	40	3.625	3.906
32–36 weeks, initial care in NICU, LNU or SCBU	0.7272	1.1	1.073	0.0365	40	2.424	2.600
≥ 37 weeks, initial care in NICU, LNU or SCBU	0.4210	1.1	1.042	0.0341	40	2.330	2.428
Survival							
All infants	0	1.1	1.000	0.0054	40	1.211	1.211
< 28 weeks, initial care in NICU	0.2264	1.1	1.023	0.0189	40	1.737	1.776
< 32 weeks, initial care in NICU or LNU	0.3278	1.1	1.033	0.0126	40	1.491	1.540
32–36 weeks, initial care in NICU, LNU or SCBU	0.3865	1.1	1.039	0.0019	40	1.074	1.116
≥ 37 weeks, initial care in NICU, LNU or SCBU	0	1.1	1.000	0.0026	40	1.101	1.101
Necrotising enterocolitis							
< 28 weeks, initial care in NICU	0.0944	1.1	1.009	0.0116	40	1.452	1.466
28–31 weeks, initial care in NICU or LNU	0.1707	1.1	1.017	0.0060	40	1.234	1.255
< 32 weeks, initial care in NICU or LNU	0.2042	1.1	1.020	0.0079	40	1.308	1.335
Brain injury							
< 28 weeks, initial care in NICU	0.1647	1.1	1.016	0.0119	40	1.464	1.488
< 32 weeks, initial care in NICU or LNU	0.2888	1.1	1.029	0.0077	40	1.300	1.338
32–36 weeks, initial care in NICU, LNU or SCBU	0.4538	1.1	1.045	0.0016	40	1.062	1.111
≥ 37 weeks, initial care in NICU, LNU or SCBU	0.3972	1.1	1.040	0.0078	40	1.304	1.356
Death or bronchopulmonary dysplasia							
< 28 weeks, initial care in NICU	0.4222	1.1	1.042	0.0108	40	1.421	1.481
28-31wks, initial care in NICU or LNU	0.3976	1.1	1.040	0.0195	40	1.761	1.830
< 32wks, initial care in NICU or LNU	0.5336	1.1	1.053	0.0440	40	2.716	2.861

*NICU* is neonatal intensive care unit, *LNU* local neonatal unit, *SCBU* is special care baby unit

N.B. Gestational age and care level strata are not mutually exclusive groups

## Discussion

We report marginal, unadjusted intracluster correlation coefficients for core and common neonatal outcomes with high precision and in subgroups relevant to neonatal trials. As expected, cluster estimates in neonatal care vary between outcomes and by population characteristics such as gestational age and the level of the neonatal unit delivering care; these result in a range of design effects and consequent inflation of sample size to account for clustering in neonatal trials. This study is unique in using national data recorded as part of routine care to estimate ICC for a wide range of outcomes; this large dataset has resulted in precise estimates with narrow confidence intervals for key outcomes based on empirical data and robustly defined core outcome sets [12, 13]. Intracluster correlation coefficients were estimated for common gestational age and neonatal unit level of care groups, making them appropriate for use in a range of neonatal trial situations. These data will inform and improve the design of individually and cluster randomised neonatal trials.

There is a paucity of published data describing neonatal cluster estimates. Rysavy et al. [21] presented neonatal unit cluster estimates ranged from 0.02 to 0.13 among preterm infants admitted to units in the Institute of Child Health and Human Development Neonatal Research Network. Although these are estimated from a different population to the NNRD, the estimates are in the same ranges. Additionally, and consistent with our results, Rysavy et al. [21] also found an inverse relationship in the magnitude of ICC estimates with gestational age.

Yelland et al. [22] estimated ICCs for multiple births from four completed perinatal trials: the DINO trial (*n* = 657), the BCD trial (*n* = 319), the TTB trial (*n* = 470) and the N3RO trial (*n* = 1273). The ICCs were estimated on a range of outcomes including death or bronchopulmonary dysplasia (BPD) and necrotising enterocolitis (NEC) and identified large differences between the ICCs for different outcomes. Intracluster correlation coefficients for death/BPD were similar although not identical to those we report: 0.33 95% CI (0.17, 0.49) in a trial with infants < 29 weeks gestational age compared to our finding of 0.42 95% CI (0.37, 0.48) for infants < 28 weeks gestational age. Necrotising enterocolitis was defined as ‘proven NEC’ in the trials analysed by Yelland et al., with the ICC reported as 0.17 95% CI (−0.04, 0.95) compared to a more precisely estimated 0.21 95% CI (0.17, 0.24) in our study. NEC for very preterm infants was comparable between Yelland et al., 0.08 (−0.07, 0.42) for infants < 29 weeks gestational age and 0.09 95% CI (0, 0.19) for infants < 28 weeks gestational age in this study. The wide confidence intervals reported by Yelland et al. highlight the limitations of using relatively small datasets and relatively few clusters from clinical trials; we build on this previous work by reporting similar ICC with much greater precision. A separate study by Yelland et al. presents the ICC estimates for multiple births from combining the infant level data from 12 preterm trials. They report some ICCs to be much larger than our study found, particularly for mortality (0.66 95% CI (versus 0.2264 in this study) and brain injuries (median ICC for intraventricular haemorrhage of 0.36 versus 0.1647 in this study). Other variations between estimates were seen for bronchopulmonary dysplasia (0.51 versus 0.4222 in this study) and necrotising enterocolitis (0.13 versus 0.09144 in this study). The estimates from Yelland et al. [23] differ from this study as they have been produced using adjusted log binomial models, which account for the effects of both trial treatment group and for known confounders. This model was not chosen for our analysis since the focus of the study was unadjusted estimates which are easily computed using ANOVA for both continuous and binary outcomes.

Large ICCs are due to larger between-cluster variations when compared to within-cluster variations. We find that the ICC for length of neonatal unit stay and other outcomes that are more closely related to unit practice are high for all stratified groups, indicating this outcome is more variable between units, potentially because practice within units may be guided by unit or regional policy. Other disease outcomes, such as mortality and brain injury, have much smaller ICCs implying that variations between clusters are small, consistent with a pattern well described in other clinical fields. ICCs are larger for mother-level estimates compared to neonatal unit-level estimates which is also to be expected, since multiples share genetics, their intrauterine environment as well as many other factors associated with neonatal outcomes such as socioeconomic deprivation. Interestingly, the ICC estimates for death or BPD had the inverse relationship with gestational age compared to other outcomes such that ICCs for both neonatal unit and mother levels were both smaller for more preterm infants. This effect may be explained by the high prevalence of death or BPD at the lower gestational age groups which would translate into less variation between clusters. In addition, since BPD is defined by receipt of treatment (respiratory support at 36 corrected gestational weeks), this outcome may be more prone to neonatal unit cluster variation than other clinical outcomes.

To illustrate the impact of different ICCs on sample size, we present the design effect for an example cluster trial; both the ICC and average cluster size are needed to calculate the design effect, and it is important to note that a small ICC will still have a substantial design effect with larger average cluster sizes. Cluster-appropriate analysis is recommended whenever clusters are present in the data; multiple births are common in preterm trials and can constitute up to 30% or more of the total trial population. In such trials the average cluster sizes and design effects are small when estimating the ICC on the level of mothers, but the benefit of exercising good practice and ensuring that the trial is suitably powered outweighs any additional challenges relating to the marginal increase in sample size.

Cluster estimates on the level of the mother are intended to be interpreted as the effects when both singletons and multiple births are present in trial datasets, rather than solely the effect of multiple births. It was chosen this way in order to estimate population-level ICCs which would be directly applicable to neonatal trial design.

Strengths of this study include use of a large, real-world, routinely recorded dataset resulting in ICCs estimated with high precision for multiple core and common neonatal outcomes, with analyses stratified by populations and cluster levels. Furthermore, we illustrate the impact of different ICCs on sample size through the design effect; a limitation of this aspect is that the design effects assume equal cluster sizes which do not hold for every trial design, especially when there is missing outcome data or when centres recruit more participants than others. In these instances, the design effect needs to be calculated with care as unequal cluster sizes require a larger sample size in order to preserve power [24, 25]. We suggest future planned neonatal cluster analyses use the data we present, rather than data from individual studies, given the magnitude of this dataset. Limitations of this work include the assignment of neonatal unit level as that of the first provider; this may have resulted in incorrect outcomes assignment in the case of infants transferred soon after birth, and further work could evaluate this against assigning the neonatal unit cluster to the longest provider; however, in practice, infants are commonly recruited to a study shortly after birth which is most likely to occur at the first provider of care. First provider may not be the neonatal unit of randomisation for trials with late interventions, and as such, these ICC estimates may be less applicable. Cluster estimates for the neonatal network level were not calculated as trials seldom, if not never, conduct cluster trials where entire neonatal networks are randomised.

A further limitation to the ICC estimates is the data accuracy and completeness of data in the NNRD; however, these should be mitigated due to the previously validated nature of this dataset for the outcomes that we focused on [8]. In addition, the inability to distinguish between missing data and non-cases in outcomes in the NNRD potentially allows for under-reporting which may have deflated the ICC estimates and is a main limitation of using routinely collected data. The estimates are unadjusted estimates and are not estimates of unit practice variation; therefore, care should be taken to avoid using the estimates as crude neonatal unit level practice variation. Care should be taken when using the brain injury outcome as it is a very broad definition that may not be appropriate to use in all trials. Similarly, length of stay is defined among survivors only, as it eliminates a main intercurrent event; however, this definition may not be useful to trialists interested in integrating death as a composite outcome.

## Conclusions

Neonatal studies should account for clustering at both the design and analysis stages. We present population-level estimates of ICC for commonly reported neonatal outcomes and trial populations to enable improved neonatal trial design and reporting.

For the purpose of open access, the author has applied a ‘Creative Commons Attribution (CC BY) licence to any Author Accepted Manuscript (AAM) version arising’.

## Data Availability

Data may be obtained from a third party and are not publicly available. The National Neonatal Research Database (NNRD) has been created through the collaborative efforts of neonatal services across the country to be a national resource. The NNRD was developed and is maintained and managed at the Neonatal Data Analysis Unit (NDAU) at the Chelsea and Westminster National Health Service (NHS) Foundation Trust campus of Imperial College London, led by Professor Neena Modi. Researchers, clinicians, managers, commissioners and others are welcome to use the NNRD and NNRD-AI. The NDAU receives no core funding to support the NNRD or NNRD-AI. For this reason, a charge is necessary to cover the costs of maintaining and developing the NNRD/NNRD-AI, data transfer, extraction, cleaning and storage, and any analyses requested. A steering board provides oversight of the NNRD/NNRD-AI. The steering board does not provide peer review but ensures the request is legitimate, feasible and in the interests of patients or the public. If the request is approved, the applicant is notified and asked to obtain necessary regulatory approvals. Applicants are asked to confirm that research findings will be published. On confirmation of regulatory approval, all eligible neonatal units are sent a copy of the research protocol, inviting them to participate. In the case of nationally commissioned service evaluations, all neonatal units are notified of the request by the lead organisation. These processes ensure that neonatal units are able to decline participation in research if they wish and are fully informed about national initiatives. If you are thinking of using the NNRD or NNRD-AI for research or health services audit or evaluations, please first read the information available from [https://www.imperial.ac.uk/neonatal-data-analysis-unit/neonatal-data-analysis-unit/utilising-the-nnrd]carefully, follow the process shown and complete the Health Data Research UK data access request ([https://healthdatagateway.org/en/dataset/619]]).

## Appendix

**Table:** Cluster estimates for mothers for binary outcomes; data from National Neonatal Research Database 2016-2020

Mother Cluster Estimates for Binary Outcomes	Missing Outcome Data n(%)^1^	N	Mean cluster size	Summary Statistics n (%)	Within Cluster SD	ICC	ICC 95% Confidence Interval
Survival (*n*=403,026) ^2^							
All infants	0 (0)	403,026	1.08	398,788 (98.95)	0.13	0	(0, 0.0153)
Gestational age and levels of care groups							
<28 weeks, initial care in NICU	0 (0)	7,616	1.13	6,102 (80.12)	0.35	0.2264	(0.1571, 0.2956)
<32 weeks, initial care in NICU or LNU	0 (0)	31,028	1.16	28,498 (91.85)	0.22	0.3278	(0.3003, 0.3553)
32-36 weeks, initial care in NICU, LNU or SCBU	0 (0)	117,365	1.14	116,683 (99.42)	0.06	0.3865	(0.3727, 0.4003)
≥37 weeks, initial care in NICU, LNU or SCBU	0 (0)	252,471	1.03	251,592 (99.65)	0.07	0	(0, 0.0336)
Necrotising enterocolitis (*n*=32,310) ^3^							
All infants	6,400 (19.81)	25,910	1.15	1,436 (5.54)	0.20	0.2021	(0.1655, 0.2388)
Gestational age and levels of care groups							
<28 weeks, initial care in NICU	1,396 (19.27)	5,848	1.12	693 (11.85)	0.31	0.0944	(0, 0.1916)
28-31 weeks, initial care in NICU or LNU	4,281 (20.43)	16,674	1.16	485 (2.91)	0.15	0.1707	(0.1253, 0.2162)
<32 weeks, initial care in NICU or LNU	6,003 (19.83)	24,263	1.15	1,362 (5.61)	0.21	0.2042	(0.1666, 0.2417)
Severe necrotising enterocolitis (*n*=33,146) ^4^							
Gestational age and levels of care groups							
<28 weeks, initial care in NICU	-	7,616	1.13	676 (8.88)	0.28	0.0288	(0, 0.1135)
28-31 weeks, initial care in NICU or LNU	-	21,167	1.17	275 (1.30)	0.11	0.0972	(0.0549, 0.1395)
<32 weeks, initial care in NICU or LNU	-	31,028	1.16	1,126 (3.63)	0.17	0.1260	(0.0914, 0.1606)
Death or bronchopulmonary dysplasia (*n*=31,805) ^5^							
Gestational age and levels of care groups							
<28 weeks, initial care in NICU	37 (0.52)	7,034	1.13	5,375 (76.41)	0.32	0.4222	(0.3658, 0.4785)
28-31 weeks, initial care in NICU or LNU	116 (0.57)	20,283	1.17	4,014 (19.79)	0.31	0.3976	(0.3671, 0.4281)
<32 weeks, initial care in NICU or LNU	168 (0.57)	29,426	1.16	10,819 (36.77)	0.33	0.5336	(0.5131, 0.5540)
Brain injury (*n*=402,764) ^6^							
Gestational age and levels of care groups							
<28 weeks, initial care in NICU	-	6,677	1.13	1,327 (19.87)	0.37	0.1647	(0.0905, 0.2388)
<32 weeks, initial care in NICU or LNU	-	27,139	1.16	2,786 (10.27)	0.26	0.2888	(0.2599, 0.3177)
32-36 weeks, initial care in NICU, LNU or SCBU	-	117,329	1.14	1,295 (1.10)	0.08	0.4538	(0.4414, 0.4662)
≥37 weeks, initial care in NICU, LNU or SCBU	-	252,286	1.03	5,301 (2.10)	0.11	0.3972	(0.3767, 0.4177)

NICU is neonatal intensive care unit, LNU is local neonatal unit and SCBU is special care baby unit

^1^Fields indicated by “-“ refer to data collected via a checkbox indicating presence of condition. Therefore, it is not possible to distinguish between missing data and presence of no condition

^2^Cluster analysis includes 54 NICUs, 83 LNUs, 47 SCBUs and 372,595 mothers

^3^Cluster analysis includes 54 NICUs, 83 LNUs, 47 SCBUs and 22,544 mothers

^4^Cluster analysis includes 54 NICUs, 83 LNUS, 47 SCBUs and 28,500 mothers

^5^Cluster analysis includes 54 NICUs, 83 LNUs, 47 SCBUs and 27,137 mothers

^6^Cluster analysis includes 54 NICUs, 83 LNUs, 47 SCBUs and 372,356 mothers

N.B. Gestational age and care level strata are not mutually exclusive groups

**Table:** Cluster estimates for mothers for continuous outcomes; data from National Neonatal Research Database 2016-2020

		N	Summary Statistics				ICC	ICC 95% Confidence Interval
	Missing Outcome Data n(%)^1^		Mean cluster size	Min, Max	Median (25^th^, 75^th^ centile)	Within Cluster SD		
Days in intensive care (*n*=402,811)^2^								
All infants	-	402,811	1.08	0, 321	0 (0, 0)	6.46	0.1320	(0.1186, 0.1454)
Gestational age and levels of care groups								
<28 weeks, initial care in NICU	-	7,615	1.13	0,277	20 (11, 36)	20.08	0.3380	(0.2778, 0.3981)
<32 weeks, initial care in NICU or LNU	-	30,969	1.16	0,321	8 (3, 16)	12.40	0.5412	(0.5218, 0.5607)
32-36 weeks, initial care in NICU, LNU or SCBU	-	117,329	1.14	0,254	0 (0, 0)	3.07	0.4427	(0.4301, 0.4553)
≥37 weeks, initial care in NICU, LNU or SCBU	-	252,286	1.03	0,180	0 (0, 0)	1.74	0.5129	(0.4963, 0.5295)
Days on ventilation (*n*=402,811)^2^								
All Infants	-	402,811	1.08	0,297	0 (0, 0)	4.75	0.0975	(0.0836, 0.1114)
Gestational age and levels of care groups								
<28 weeks, initial care in NICU	-	7,615	1.13	0,273	10 (3, 27)	15.62	0.4570	(0.4068, 0.5071)
<32 weeks, initial care in NICU or LNU	-	30,969	1.16	0,297	1 (0, 6)	9.36	0.5957	(0.5784, 0.6130)
32-36 weeks, initial care in NICU, LNU or SCBU	-	117,329	1.14	0,237	0 (0, 0)	2.10	0.3691	(0.3549, 0.3832)
≥37 weeks, initial care in NICU, LNU or SCBU	-	252,286	1.03	0,175	0 (0, 0)	1.32	0.5059	(0.4890, 0.5227)
Days on parenteral nutrition (*n*=402,811)^2^								
All infants	-	402,811	1.08	0,283	0 (0, 0)	6.28	0.0125	(0, 0.0276)
Gestational age and levels of care groups								
<28 weeks, initial care in NICU	-	7,615	1.13	0,271	15 (9, 25)	19.18	0.2313	(0.1624, 0.3002)
<32 weeks, initial care in NICU or LNU	-	30,969	1.16	0, 283	9 (5, 15)	12.16	0.4291	(0.4054, 0.4529)
32-36 weeks, initial care in NICU, LNU or SCBU	-	117,329	1.14	0,250	0 (0, 0)	3.51	0.3779	(0.3640, 0.3919)
≥37 weeks, initial care in NICU, LNU or SCBU	-	252,286	1.03	0,183	0 (0, 0)	1.57	0.3669	(0.3454, 0.3884)
Days with central line (*n*=402,811)^2^								
All infants	-	402,811	1.08	0,314	0 (0, 0)	6.77	0	(0, 0.0153)
Gestational age and levels of care groups								
<28 weeks, initial care in NICU	-	7,615	1.13	0,267	15 (9, 27)	20.23	0.2272	(0.1580, 0.2964)
<32 weeks, initial care in NICU or LNU	-	30,969	1.16	0,314	9 (4, 15)	12.95	0.4292	(0.4055, 0.4530)
32-36 weeks, initial care in NICU, LNU or SCBU	-	117,329	1.14	0,250	0 (0, 0)	3.96	0.3468	(0.3322, 0.3614)
≥37 weeks, initial care in NICU, LNU or SCBU	-	252,286	1.03	0,191	0 (0, 0)	2.13	0.2580	(0.2328, 0.2831)
Days on neonatal unit (*n*=396,718) ^3^								
All infants	6,308 (1.57)	396,718	1.08	[0,857]	4 (2, 10)	12.56	0.5714	(0.5644, 0.5784)
Gestational age and levels of care groups								
<28 weeks, initial care in NICU	1,808 (23.74)	5,808	1.11	[0,369]	95 (76, 116)	21.89	0.6178	(0.5728, 0.6629)
<32 weeks, initial care in NICU or LNU	3,299 (10.66)	27,721	1.16	[0,370]	52 (37, 78)	16.26	0.7762	(0.7656, 0.7868)
32-36 weeks, initial care in NICU, LNU or SCBU	1,365 (1.16)	116,000	1.14	[0,325]	9 (3, 16)	6.00	0.7272	(0.7207, 0.7336)
≥37 weeks, initial care in NICU, LNU or SCBU	1,473 (0.58)	250,998	1.03	[0,857]	2 (1, 5)	4.86	0.4210	(0.4012, 0.4408)

¹ Fields indicated by “-“ refer to data collected via a checkbox indicating presence of condition. Therefore, it is not possible to distinguish between missing data and presence of no condition

^2^Cluster analysis includes 54 NICUs, 83 LNUs, 44 SCBUs and 372,402 unique mothers

^3^Cluster analysis includes 54 NICUs, 83 LNUs, 47 SCBUs and 367,675 unique mothers

N.B. Gestational age and care level strata are not mutually exclusive groups

**Table:** List of all included units in the complete analysis of the National Neonatal Research Database

England	Aintree Centre For Women’s Health
	Airedale General Hospital
	Alexandra Hospital
	Alnwick Infirmary
	Andover War Memorial Hospital
	Arrowe Park Hospital
	Ashurst Hospital
	Barking Birth Centre
	Barking Hospital
	Barnet Hospital
	Barnsley District General Hospital
	Barts Health NHS Trust
	Basildon Hospital
	Basingstoke & North Hampshire Hospital
	Bassetlaw District General Hospital
	Bedford Hospital
	Bethlem Royal Hospital
	Birmingham Children's Hospital
	Birmingham Heartlands Hospital
	Birmingham Women's Hospital
	Blackburn Birth Centre
	Blackburn Royal Infirmary
	Bradford Royal Infirmary
	Bridgnorth Maternity Unit
	Bridgwater Hospital
	Brighton General Hospital
	Bristol Children's Hospital
	Bristol Royal Infirmary
	Broomfield Hospital
	Calderdale Royal Hospital
	Chelmsford & Essex Hospital
	Chelsea & Westminster Hospital
	Cheltenham General Hospital
	Chesterfield & North Derbyshire Royal Hospital
	Chippenham Community Hospital
	Chipping Norton Hospital
	Chorley & South Ribble Hospital
	City Hospital, Birmingham
	Clacton And District Hospital
	Colchester General Hospital
	Conquest Hospital
	Cossham Hospital
	Countess Of Chester Hospital
	Crowborough Birthing Centre
	Croydon University Hospital
	Cumberland Infirmary
	Darent Valley Hospital
	Darlington Memorial Hospital
	Derriford Hospital
	Dewsbury & District Hospital
	Diana Princess Of Wales Hospital
	Doncaster And Bassetlaw Hospitals NHS Foundation Trust
	Doncaster Royal Infirmary
	Dorset County Hospital
	Ealing Hospital
	East Lancashire Hospitals NHS Trust
	East Surrey Hospital
	Eastbourne District General Hospital
	Edgware Community Hospital
	Epsom General Hospital
	Fairfield General Hospital
	Frimley Park Hospital
	Frome Victoria Hospital
	Furness General Hospital
	General Surgery (Huddersfield Royal Infirmary)
	George Eliot Hospital
	Glenfield Hospital
	Gloucestershire Royal Hospital
	Good Hope Hospital
	Goole & District Hospital
	Great Western Hospital
	Guy's & St Thomas' Hospital
	Hammersmith Hospital
	Harrogate District Hospital
	Heatherwood And Wexham Park Maternity
	Helston Hospital
	Hereford County Hospital
	Hereford County Maternity
	Hexham General Hospital
	Hillingdon Hospital
	Hinchingbrooke Hospital
	Homerton Hospital
	Honiton Hospital
	Huddersfield Royal Infirmary
	Hull Royal Infirmary
	Imperial College Healthcare NHS Trust (Maternity)
	Ipswich Hospital
	James Cook University Hospital
	James Paget Hospital
	Kettering General Hospital
	Kidderminster Treatment Centre
	King George Hospital
	King's College Hospital
	King's Mill Hospital
	Kingston Hospital
	Lancashire Women & Newborn Centre
	Leeds Neonatal Service
	Leicester Neonatal Service
	Leighton Hospital
	Leeds General Infirmary Fetal Medicine and Antenatal Unit
	Lincoln County Hospital
	Lister Hospital
	Liverpool Women's Hospital
	Ludlow Maternity
	Luton & Dunstable Hospital
	Macclesfield District General Hospital
	Maidstone Birthing Centre
	Maidstone General Hospital
	Manchester Royal Infirmary
	Manor Hospital
	Medway Maritime Hospital
	Mid Staffs General Hospital
	Milton Keynes Foundation Trust Hospital
	Morriston Hospital
	Musgrove Park Hospital
	New Cross Hospital
	New Forest Birthing Centre
	Newham General Hospital
	Newton Abbot Hospital
	Norfolk & Norwich University Hospital
	North Devon District Hospital
	North Manchester General Hospital
	North Middlesex University Hospital
	North Staffordshire Royal Infirmary
	Northampton General Hospital
	Northern General Hospital
	Northumbria Specialist Emergency Care Hospital
	Northwick Park Hospital
	Nottingham City Hospital
	Nottingham University Hospital (Queen’s Medical Centre)
	Okehampton Community Hospital
	Ormskirk District General Hospital
	Oxford University Hospitals, Horton Hospital
	Oxford University Hospitals, John Radcliffe Hospital
	Paulton Memorial Hospital
	Peterborough City Hospital
	Pilgrim Hospital
	Pinderfields General Hospital
	Poole Hospital NHS Foundation Trust
	Portland Hospital
	Princess Alexandra Hospital
	Princess Anne Hospital
	Princess Royal Hospital
	Princess Royal Hospital, Telford
	Princess Royal University Hospital
	Queen Alexandra Hospital
	Queen Charlotte's Hospital
	Queen Elizabeth Hospital, Birmingham
	Queen Elizabeth Hospital, Gateshead
	Queen Elizabeth Hospital, King's Lynn
	Queen Elizabeth Hospital, Woolwich
	Queen Elizabeth The Queen Mother Hospital
	Queen's Hospital, Burton On Trent
	Queen's Hospital, Romford
	RAF Lakenheath
	Rosie Maternity Hospital, Addenbrookes
	Rotherham District General Hospital
	Royal Albert Edward Infirmary
	Royal Berkshire Hospital
	Royal Bolton Hospital
	Royal Bournemouth Hospital
	Royal Brompton Hospital
	Royal Cornwall Hospital
	Royal Derby Hospital
	Royal Devon & Exeter Hospital
	Royal Hampshire County Hospital
	Royal Lancaster Infirmary
	Royal Liverpool University Hospital
	Royal Oldham Hospital
	Royal Preston Hospital
	Royal Stoke University Hospital
	Royal Surrey County Hospital
	Royal Sussex County Hospital
	Royal United Hospital
	Royal Victoria Infirmary
	Russells Hall Hospital
	Salford Royal Hospital
	Salisbury District Hospital
	Samuel Johnson Community Hospital
	Samuel Johnson Community Maternity Hospital
	Scarborough General Hospital
	Scunthorpe General Hospital
	Solihull Hospital
	South Tyneside District Hospital
	Southampton General Hospital
	Southend Hospital
	Southmead Hospital
	St Austell Community Hospital
	St George's Hospital
	St Helier Hospital
	St Mary's Birthing Centre
	St Mary's Hospital, Iow
	St Mary's Hospital, London
	St Mary's Hospital, Manchester
	St Michael's Hospital
	St Peter's Hospital
	St Richard's Hospital
	Stepping Hill Hospital
	Stockport Maternity
	Stoke Mandeville Hospital
	Stroud General Hospital
	Sunderland Royal Hospital
	Tameside General Hospital
	The Grange Surgery
	The Jessop Wing, Sheffield
	The Royal Free Hospital
	The Royal London Hospital
	Tiverton And District Hospital
	Torbay Hospital
	Trowbridge Community Hospital
	Tunbridge Wells Hospital
	University College Hospital
	University Hospital Coventry
	University Hospital Lewisham
	University Hospital Of North Durham
	University Hospital Of North Tees
	Victoria Hospital, Blackpool
	Victoria Medical Centre
	Wallingford Community Hospital
	Wantage Hospital
	Warrington Hospital
	Warwick Hospital
	Watford General Hospital
	West Cumberland Hospital
	West Middlesex University Hospital
	West Suffolk Hospital
	Westmorland General Hospital
	Weston General Hospital
	Wexham Park Hospital
	Whipps Cross University Hospital
	Whiston Hospital
	Whittington Hospital
	William Harvey Hospital
	William Julien Courtauld Hospital
	Worcestershire Royal Hospital
	Worthing Hospital
	Wycombe Hospital
	Wythenshawe Hospital
	Yeovil District Hospital
	York District Hospital
Ireland	Coombe Women's Hospital, Dublin
Northern Ireland	Altnagelvin
	Antrim Hospital
	Craigavon Area Hospital
	Royal Jubilee Maternity Belfast
	Royal Maternity Hospital, Belfast
	Uist & Barra Hospital
Scotland	Aberdeen Maternity Hospital
	Arbroath Infirmary
	Ayrshire Maternity Unit, Crosshouse
	Balfour
	Balfour Hospital Maternity Unit
	Belford, Fort William
	Borders General Hospital, Melrose
	Caithness General Wick
	Cowal Community Hospital
	Dr Gray's Hospital, Elgin
	Dr Mackinnon Memorial Hospital
	Dumfries & Galloway Royal Infirmary
	Forth Valley Royal Hospital
	Galloway Community Hospital
	Gilbert Bain, Lerwick Shetland
	Golden Jubilee Hospital, Glasgow
	Inverclyde Royal Hospital
	Inverurie Community Maternity Unit
	Islay Hospital
	Kirkwall Orkney
	Lorn & Islands District General Hospital
	Mid-Argyll Community Hospital
	Monklands Hospital, Airdrie
	Montrose Royal Infirmary
	Ninewells Hospital, Dundee
	Perth Royal Infirmary
	Perth Royal Infirmary, Maternity
	Peterhead Community Hospital
	Pregnancy Support Centre – Edinburgh
	Princess Royal Maternity, Glasgow
	Raigmore Hospital, Inverness
	Royal Aberdeen Children's Hospital
	Royal Alexandra Hospital, Paisley
	Royal Hospital For Children, Glasgow
	Royal Hospital For Sick Children, Edinburgh
	Simpson Centre For Reproductive Health, Edinburgh
	St John's Hospital, Livingston
	Vale Of Leven Maternity
	Victoria Hospital, Fife
	Victoria Hospital, Rothesay
	Western Isles Hospital
	Wishaw General Hospital
Wales	Bronglais General Hospital
	Glan Clwyd Hospital
	Glangwili General Hospital
	Heath Hospital Maternity
	Llandrindod Wells Hospital
	Mlu - Neath Port Talbot
	Nevill Hall Hospital
	Prince Charles Hospital
	Princess Of Wales Hospital
	Royal Glamorgan Hospital
	Royal Gwent Hospital
	Singleton Hospital
	University Hospital Of Wales
	Withybush Hospital
	Wrexham Maelor Hospital
	Ysbyty Gwynedd
	Ysbyty Ystrad Fawr
British Crown Dependencies	Jersey General Hospital
	Nobles Hospital
	Princess Elizabeth Hospital, Guernsey

## Abbreviations

ANOVA: Analysis of variance
BAPM: British Association of Perinatal Medicine
BPD: Bronchopulmonary dysplasia
CI: Confidence interval
ICC: Intracluster correlation coefficient
LNU: Local neonatal unit
NDAU: Neonatal Data Analysis Unit
NEC: Necrotising enterocolitis
NHS: National Health Service
NICU: Neonatal intensive care unit
NNAP: National Neonatal Audit Programme
NNRD: National Neonatal Research Database
SCBU: Special care baby unit
SD: Standard deviation

## Declarations

Dr. Gale reports personal support from the United Kingdom Medical Research Council Transition Support Fellowship during the conduct of the study; grants from National Institute for Health Research, Rosetrees Foundation, Canadian Institute for Health Research, Action Medical Research outside the submitted work; grants for research outside the submitted work and personal fees to support attendance at educational events from Chiesi Pharmaceuticals; he is chair of the NIHR Research for Patient Benefit London Regional Assessment Panel.
